# Omega-3 Long-Chain Polyunsaturated Fatty Acids Intake in Children: The Role of Family-Related Social Determinants

**DOI:** 10.3390/nu12113455

**Published:** 2020-11-11

**Authors:** María Isabel Martínez-Martínez, Antoni Alegre-Martínez, Omar Cauli

**Affiliations:** 1 Frailty and Cognitive Impairment Organized Group (FROG), University of Valencia, 46010 Valencia, Spain; m.isabel.martinez@uv.es; 2 Department of Nursing, University of Valencia, 46013 Valencia, Spain; 3 Department of Biomedical Sciences, Cardenal Herrera University CEU, Avenida Seminario, s/n, 46113 Moncada, Valencia, Spain; antoni.alegre@uchceu.es

**Keywords:** fish intake, omega-3 fatty acids, nutrients, ADHD, children, diet-deficient

## Abstract

Omega-3 long-chain polyunsaturated fatty acids play a central role in neuronal growth and in the development of the human brain, since they are essential elements which depend on intake through diet to ensure an adequate amount. Fish and seafood are the main dietary sources of these fatty acids in Spain and in other countries. In order to assess the effect of the intake of common foods containing high amounts of omega-3 polyunsaturated fatty acids, a food frequency questionnaire was administered to parents of children and adolescents attending a primary school in Valencia (Spain), and the intake of dietary omega-3 such as eicosapentaenoic acid (EPA) and docosahexaenoic acid (DHA) was estimated based on their fish/seafood consumption. Low frequencies of intake were significantly (*p* < 0.05) lower for many types of fish/seafood in children compared to adolescents. 27.5% of children/adolescents did not eat lean fish or other types (19.8% of the sample did not eat fatty fish, and 71.8% did not eat smoked fish) and 20–60% of the sample consumed seafood only once–three times a month, leading to a reduced estimated intake of EPA+DHA below that recommended for both groups by public health agencies. Social aspects, such as the type of work done by mothers and their educational levels are significant factors (*p* < 0.05 in both cases) affecting children’s/adolescents’ intake of DHA+EPA. Dietary interventions to increase the consumption of fish and seafood are strongly advised, and health promotion strategies should be aimed at the family level and fight against gender disparities.

## 1. Introduction

Essential fatty acids are a fundamental and necessary nutritional contribution to the healthy development of the organism and, of all of them, omega-3 long chain polyunsaturated fatty acids (LC-PUFA) are those that have presented the healthiest effects. Several reports have shown that the contribution of O3 through diet can have cardioprotective effects, reducing the incidence of acute myocardial infarction, act as an antihypertensive, have anti-inflammatory effects, and intervene in the process of inhibiting the growth of some tumor cells in the adult population, among other benefits [1,2,3,4,5]. In children, an adequate O3 intake prevents obesity-related chronic diseases [6], and lowers the risk of allergies [7], increased visual acuity [1,8] and improved cognitive ability [5,9,10,11], in particular O3 in the form of Eicosapentaenoic Acid (EPA) and Docosahexaenoic Acid (DHA). EPA and DHA are part of the organism′s cell membrane phospholipids, but DHA has the highest presence, up to 50%, in the organ-specific membrane phospholipids such as in the retina and the cerebral cortex [12,13]. This determines the importance of O3 LC-PUFA for the correct functioning and development of these organs, as well as in the deterioration of some functions when these substances are deficient, such as impairment of brain function, lack of growth, skin lesions, loss of muscle tone and degenerative changes in some organs [2,14,15,16,17]. Several studies relate fish consumption to socio-economic conditions: in a study performed in Amsterdam in 2014, only 31.7% of participants adhered to the fish consumption guidelines (in comparison to 82.5% for the fruit guideline), and the group with the lowest income adhered to it the least (OR 0.55 compared to the high income group). However, occupational prestige was not statistically associated with its consumption [18]. In another French study, seafood intake recommendations were better followed by older people and those with a higher educational level and high occupational category, and the budget allocated to fish also increased with age and educational level [19]. In a Chinese study performed in 2007–2009, respondents consuming the least fish were individuals with low incomes and an education below primary school level [20].

The data on O3 LC-PUFA consumption from seafood in children are very scarce [16,21,22], and the only generalized and inconclusive recommendations available on the consumption of the most common O3 such as EPA and DHA, resulting mainly from consumption of fish, are those of organizations such as the United Nations Food and Agriculture Organization, the World Health Organization (FAO/WHO) and the Spanish Society for Community Nutrition [14,23,24] and their derivatives. Our eating behavior is conditioned by various socio-cultural determinants and acquires many social meanings [25,26,27]. Fish intake is heavily influenced by the social environment, the opinions of family, doctors and nutritionists, availability in the market, ease of cooking and making a good choice when purchasing [28,29]. The family has been described as the main factor encouraging the young to choose fish dishes [29,30,31]. One of the threats to fish consumption is the perception that it contains harmful substances such as mercury [32,33,34]. Although presenting information about the limits of harmful substances in fish markets increases the feeling of safety among regular fish consumers [35], high levels of fish consumption are still considered potentially harmful. In the diet of children and adolescents, the family, the parents’ jobs, their educational level and possibly whether the family is single-parent or two-parent are important factors when considering an adequate diet in the household environment. The influence of the parents’ educational style, the intake of healthy food based on communication between parents and children about which foods are more nutritious, and the children’s involvement in shopping and cooking are crucial in providing an adequate and balanced diet [36]. The relationship between the mother’s dietary practices and the child’s healthy diet increases the life satisfaction and the frequency of shared family meals, and increases the future adolescent’s well-being [37,38].

The present study was therefore designed with the following three main objectives:

(1) Evaluation of the pattern of consumption of O3 LC-PUFA by analysis of the main dietary sources of fish.
(2) Estimation of the daily intake of omega-3 long chain fatty acids (EPA+DHA).
(3) Evaluation of the influence of age, gender and body mass index and social determinants related to the family, such as the parents’ education and employment situation, on children’s O3 LC-PUFA intake.

## 2. Materials and Methods

### 2.1. Study Design

We performed a cross-sectional study in primary schools (children and adolescents aged between 6 and 12 years old) in Valencia (Spain) between 2018 and 2019. The study participants were parents of children recruited from two public sector schools without any chronic diseases (an exclusion criterion for participation in this study). Body mass index was calculated as weight in kilograms divided by the square of height in meters. BMI is age- and sex-specific for children and teens, and is often referred to as BMI-for-age. According to the international guidelines, BMI is grouped in four categories: underweight (BMI less than the 5th percentile); normal or healthy weight (5th percentile to less than the 85th percentile); overweight (85th percentile to less than the 95th percentile); and obese (equal to or greater than the 95th percentile) [39]. The social aspects were related to the parents’ level of education (primary or secondary school, university studies), work situation (unemployed/housewife; primary sector: commodities sector, agriculture, livestock, fishing, mining; secondary sector: factories and industry; or tertiary sector: services) and information about living with two parents or one. The study comprised 131 children of both sexes. The study protocol was approved by the Ethics Committee of the University of Valencia (protocol number H1397475950160).

### 2.2. Dietary Assessment

In order to estimate the diet of the children and adolescents enrolled in the study we asked parents to complete a food frequency questionnaire about their children’s diet, recording also the intake of beverages and nutritional supplement consumption if any. The questionnaire employed was a semi-quantitative food questionnaire comprising 136 food items and had been previously validated in Spain [29]. The parents were asked to estimate the portion sizes for each diet item ingested by their sons/daughters according to a visual guide [16,40,41,42] in order to improve the accuracy of these estimations. Food intake was estimated by crossing the frequency intake of any food and the portion size consumed for each food. All food records were analyzed using the Nutrition Data Systems-Research free software package (DIAL^®^, developed by the Department of Nutrition and Dietetics, Complutense University, Madrid, Spain, and validated in Spain). Nutrient intake was averaged across the two days, and normalized to intake per 1000 kcal. Energy and specific nutrient intake were calculated based on the Spanish food composition tables [43,44].

### 2.3. Estimation of Omega-3 Long Chain Polyunsaturated Fatty Acid (LC-PUFA) Intake from Fish and Seafood

Parents self-reported their children’s fish and nut consumption. Fish was defined as “any kind of fish, including fish sticks and canned tuna fish, shellfish, crustaceans and mollusks”. The participants reported: (a) how often they consumed fish (“did not eat”, “once–three times a month”, “about once a week”, “twice-four times a week”, “five-six times a week”, “once a day”); and (b) the type of fish they typically consumed.

The items in the three-day semi-quantitative food questionnaire [16,45] related to fish and seafood consumption and their omega-3 LC-PUFA contents (g/100 g of food item, as the sum of EPA+DHA) were: (a) lean fish: young hake, hake, sea bream, grouper and sole (0.62); (b) fatty fish: salmon, mackerel, tuna, Atlantic bonito and sardine (1.87); (c) cod (0.70); (d) smoked and salted fish: salmon and herring (4.44); (e) shellfish: mussels, oysters and clams (2.20); (f) seafood: shrimp, prawns and crayfish (0.90), and (g) mollusks: octopus, cuttlefish and squid (0.71). Omega-3 LC-PUFA intake was calculated as frequency x (EPA+DHA) content for each food item (fish, seafood) [16,45,46]. We estimated the intake of EPA+DHA because these fatty acids are the most common O3 in fish and seafood [23,47]. In addition, we asked the parents about the frequency of consumption of omega-3 long fatty acid supplements or omega-3 fatty acid-enriched milks. The intake of omega-3 LC-PUFA and fish consumption were adjusted for total energy intake using the residuals method proposed by Willett et al. [44].

### 2.4. Statistical Analysis

Categorical variables were represented as absolute frequencies and percentage over the total number of children/adolescents enrolled in the study. Quantitative (continuous) variables were expressed as mean ± the standard error. In the bivariate analysis, we first evaluated the normal or non-normal data distribution for quantitative variables using the Shapiro-Wilk (*n* < 50) or Kolmogorov-Smirnoff (*n* ≥ 50) test. The non-normal distribution suggested the use of non-parametric tests when comparing continuous variables across groups (categorical variables), e.g., the Mann-Whitney *U*-test (when comparing quantitative variables between two groups) and the Kruskal-Wallis test (when comparing quantitative variables between three or more groups). Correlation analysis between quantitative variables was performed using the non-parametric Spearman’s correlation test. Partial correlations were performed in order to control for the effect of intervening/confusing variables. Differences between categorical variables were evaluated with the Chi-squared test. Statistical significance was considered when *p* < 0.05. The SPSS version 24.0 statistical package (SPSS, Inc., Chicago, IL, USA) was used throughout.

## 3. Results

### 3.1. Description of the Sample

The study comprised 131 children of both sexes (39.7% female and 60.3% male) living in Valencia (Spain), which is the country’s third most populous city. The characteristics of the study sample are shown in Table 1. The sample was composed of 39.7% female and 60.3% male children. The mean BMI was 19.1 ± 3.8 (range 10.6–30.5). Weight distribution revealed that only 34.5% of the children were of normal weight, 23.7% were underweight (percentile < 5), and 42% were overweight (percentile 85–94) or obese (≥95 percentile).

As for the parents’ educational level, 21–25% had attended primary school, 37–43% had attended secondary school, and 36–37% had attended university. 29% of the mothers and only 6.1% of the fathers were unemployed. Among the parents with a job, most (both mothers and fathers) worked in the tertiary sector (around 54% for both sexes), and 16.2% of mothers and 35.1% of fathers worked in the secondary sector, whereas none (0% of the mothers) or few (4.6% of the fathers) worked in the primary sector.

### 3.2. Energy Intake and Frequency of Fish and Seafood Consumption

The reported average energy intake in our study sample was approximately 1723 kcal. Of this amount, 51% was carbohydrates, 35% was fat and 14% was protein. As for lean fish intake (including young hake, cod, hake, blackspot sea bream, goliath grouper, and common sole), 27.5% of the sample did not eat lean fish, 41.2% ate lean fish once­–three times a month, and 31.3% ate lean fish once a week or more frequently. For fatty fish intake (salmon, mackerel, tuna, bonito, sardine), 19.8% of the sample did not eat fatty fish, 64.1% ate fatty fish once–three times a month, and 16.1% ate fatty fish once a week or more frequently. For smoked fish (including smoked and salted fish such as salmon and herring), 71.8% did not eat it, 21.4% ate smoked fish once–three times a month and 6.8% ate smoked fish once a week. The intake of mollusks (including octopus, common cuttlefish, and squid) was as follows: 35.9% did not eat them, 48.9% ate them once–three times a month, and 15.2% ate them once a week or more frequently. As for their intake of crustaceans (including shrimps, prawn, and crayfish), 41.9% did not eat them, 38.8% ate crustaceans once–three times a month, and 19.4% did so once a week or more frequently. Intake of shellfish (including mussel, oyster, and clam) was as follows: 47.7% did not eat them, 38.3% ate them once–three times a month, and 14.0% ate them once a week or more frequently. 3.5 % of the sample consumed omega-3 long fatty acid supplements (capsules containing fish oil) and 13% of the sample consumed omega-3 fatty acid-enriched milks. There were no significant differences for fish and seafood intake between the sexes (*p* = 0.16 for lean fish; *p* = 0.10 for fatty fish; *p* = 0.86 for smoked fish; *p* = 0.95 for; mollusks *p* = 0.31; for crustaceans; *p* = 0.09 for shellfish; *p* = 0.72 for omega-3 long fatty acid supplements; *p* = 0.16 for omega-3 fatty acid-enriched milks). There were no significant differences for fish and seafood intake between underweight/normal weight/overweight children (*p* = 0.39 for lean fish; *p* = 0.44 for fatty fish; *p* = 0.66 for smoked fish; *p* = 0.62 for mollusks *p* = 0.31; for crustaceans; *p* = 0.39 for shellfish; *p* = 0.08 for omega-3 long fatty acid supplements; *p* = 0.62 for omega-3 fatty acid-enriched milks). By categorizing the age of the children/adolescents, we analysed the frequencies of fish/seafood intake in children (age ≤ 9) and in adolescents (age ≥ 10) based on the World Health Organization’s definition [48]. We observed a significantly higher intake of fatty and smoked fish in adolescents compared to children (*p* = 0.04 and *p* = 0.03, respectively) whereas no difference were found for the intake of other types of fish/seafood, omega-3 long fatty acid supplements or the intake of omega-3 fatty acid-enriched milks (*p* > 0.05 in all cases).

### 3.3. Fish and Seafood Intake and Parent’s Social Aspects

There were no differences in the frequency of fish and seafood intake in terms of living with two parents or one parent (*p* = 0.67 for lean fish; *p* = 0.66 for fatty fish; *p* = 0.84 for smoked fish; *p* = 0.16 for mollusks; *p* = 0.95 for shellfish; *p* = 0.69 for crustaceans; *p* = 0.87 for omega-3 long fatty acid supplements; *p* = 0.33 for omega-3 fatty acid-enriched milks) except for shellfish intake (*p* = 0.002): 40.6% of children living with both parents consumed shellfish once–three times a month compared to 27.3% of children living only with one parent.

**Figure 1:**
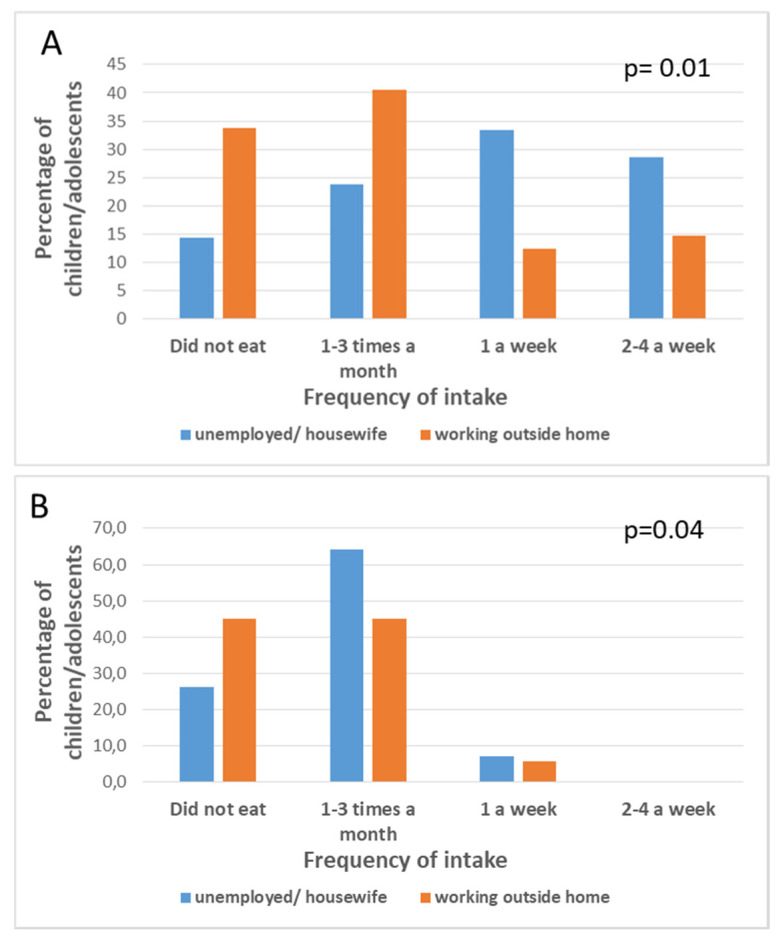
Frequency of intake (expressed as percentage of children/adolescents in the sample) for lean fish (**A**) and fatty fish (**B**) in children/adolescents whose mothers are unemployed /housewife or work outside home.

There was no significant association between the father’s type of work and the children’s fish intake (*p* = 0.94 for lean fish; *p* = 0.92 for fatty fish; *p* = 0.84 for smoked fish; *p* = 0.94 for mollusks *p* = 0.19 for crustaceans; *p* = 0.90 for omega-3 long fatty acid supplements; *p* = 0.20 for omega-3 fatty acid-enriched milks), except for shellfish intake (*p* = 0.01). However, there were significant associations between the mother’s work and the children’s fish intake (*p* = 0.01 for lean fish; *p* = 0.04 for fatty fish) (Figure 1).

No significant associations were found for other types of seafood (*p* = 0.44 for shell fish; *p* = 0.78 for mollusks; *p* = 0.98 for crustaceans; *p* = 0.74 for omega-3 long fatty acid supplements; *p* = 0.34 for omega-3 fatty acid-enriched milks). No significant associations were observed between the father’s level of education and fish intake in children (*p* > 0.05 for all types of fish/seafood) or between mother’s level of education and fish intake among children (*p* > 0.05 for all types of fish/seafood).

### 3.4. Estimation of Omega-3 Long-Chain Polyunsaturated Fatty Acids Intake

**Figure 2:**
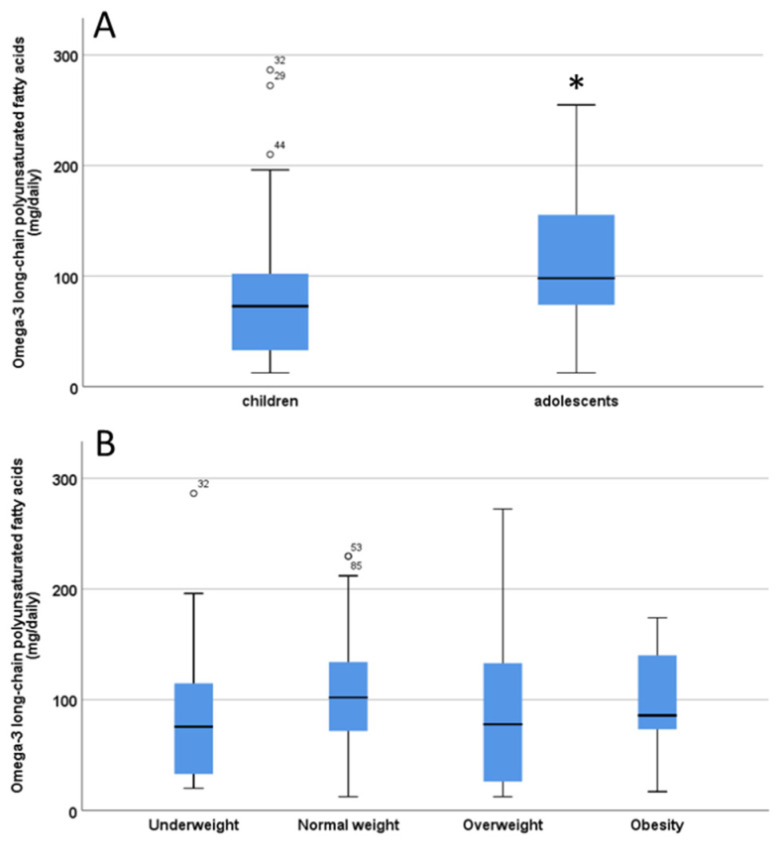
Estimation of omega-3 long chain polyunsaturated fatty acids (LC-PUFA) intake from fish/seafood in children and adolescent (**A**) and body weight categories (**B**). The significant difference in Panel A is indicated by the asterisk * *p* < 0.05. ° indicate outliers defined as a data points located outside the whiskers of the box plot.

The estimated ingestion of omega-3 long-chain polyunsaturated fatty acids (O3) from fish/seafood (EPA+DHA) in the diet was 101.6 ± 5.9 mg/day (range 12.4–286.4). There were no significant differences (*p* = 0.10) in O3 intake between males and females (females: 99.3 ± 8.3 versus males: 99.2 ± 6.2 mg/day). There was a significant difference (*p* = 0.02) in O3 intake between children and adolescents (children: 84.9 ± 8.1 versus adolescents: 110.4 ± 6.7) (Figure 2A). No significant differences in O3 intake were found for different weight categories (underweight, normal weight, over weight /obesity, *p* = 0.11) (Figure 2B).

**Figure 3:**
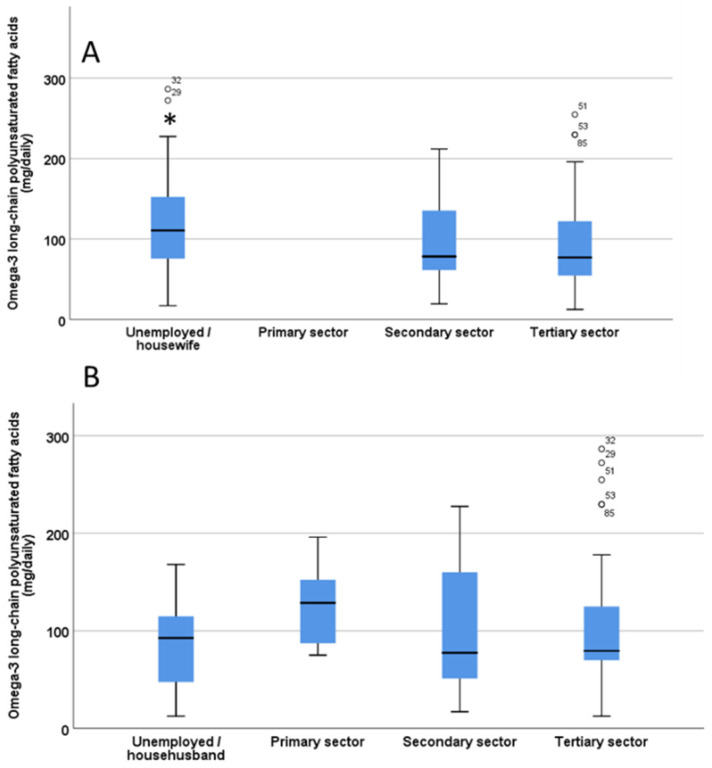
Estimation of omega-3 long chain polyunsaturated fatty acids (LC-PUFA) intake from fish/seafood in children/adolescents based on the mother’s work (**A**) and father’s work (**B**). The significant difference in Panel A is indicated by the asterisk * *p* < 0.05. ° indicate outliers defined as a data points located outside the whiskers of the box plot.

There were significant differences (*p* = 0.02) in the O3 intake of children/adolescents based on mother’s type of work, being significantly higher when the mother was unemployed or a housewife (Figure 3A). In contrast, no significant differences were found in the O3 intake of children/adolescents based on the father’s work (*p* = 0.28, Figure 3B).

**Figure 4:**
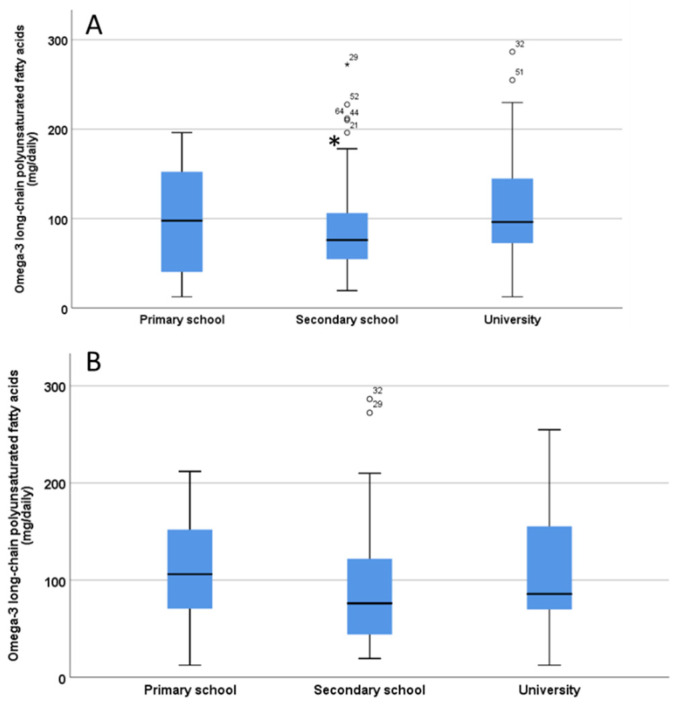
Estimation of omega-3 long chain polyunsaturated fatty acids (LC-PUFA) intake from fish/seafood in children/adolescents based on the level of education of the mothers (**A**) and fathers (**B**). The significant difference in Panel A is indicated by the asterisk * *p* < 0.05. ° indicate outliers defined as a data points located outside the whiskers of the box plot.

No significant differences (*p* = 0.90) were found in the O3 intake of children/adolescents between children living with one parent and those living with both parents. There were significant differences in the O3 intake of children/adolescents based on the mother’s educational level (*p* = 0.03), as children whose mothers had completed secondary school education had a lower intake of O3 (Figure 4A), while in contrast no significant differences (*p* = 0.19) regarding fathers’ educational level were found (Figure 4B).

## 4. Discussion

Fish/seafood is an important dietary source of omega-3 long-chain polyunsaturated fatty acids such as EPA and DHA, which are essential for a proper metabolism [31,47,49,50]. The results of our study revealed a low frequency of fish intake, as 27.5% of children/adolescents did not eat lean or other types of fish (19.8% of the sample did not eat fatty fish and 71.8% did not eat smoked fish) and around 20%–60% of the sample consumed seafood only once–three times a month, leading to a reduced intake of essential omega-3 long chain fatty acids such as DHA and EPA. The European Food Safety Authority made a recommendation of 250 mg/day of EPA + DHA in the pediatric population [51]. The Food and Agriculture Organization and the World Health Organization recommends an intake of EPA+DHA of about 100–200 mg/day for children aged 2–6 years and 200–250 mg/day for those older [52,53]. To our knowledge, no studies have examined whether the intake of fish/seafood and related omega-3 long chain fatty acids EPA and DHA (expressed as mg/day) in the diet is adequate in children/adolescents without a diagnosed disease/disorder. Our study shows worrying results in the form of a very low intake of fish/seafood and related EPA+DHA among Spanish children/adolescents, compared to the amounts recommended by the public health organizations (50–60% lower than the recommended daily dose). Similar findings have also emerged from a recent French population-based study of children (3–10 years) and adolescents (11–17 years) [54,55]. The estimated intake of EPA+DHA was not affected by sex or body mass weight but it was affected by age. In fact, we observed a significantly higher intake of fatty and smoked fish in adolescents compared to children, which was associated with a significantly higher estimated intake of O3 in adolescents. These results suggested that preferences for seafood/fish increased during development, which would lead to a better nutritional profile in adolescence. However, when we looked at a distribution of the estimation of O3 intake we still found 40% of adolescents whose intake was below the levels recommended by current nutritional guidelines. Our results confirmed the findings obtained for Norwegian and Dutch adolescents, which showed an insufficient status of important nutrients such as O3 derived from fish consumption [9,11,56]. This is a cause for major concern, as both Norway and Spain are among the leading fish consuming countries in the world, and this low fish intake may be even worse in other countries which consume less seafood than Spain or Norway [29]. The recommendation for fish intake in the United States is twice per week, avoiding species with high mercury content [57]. The recommendation in the United Kingdom is for two meals of fish per week, of which one should be a fatty fish [58]. Reduced fish intake (and related reduced intake of O3 such as EPA and DHA) during childhood and adolescence may be associated with lower processing speed, a poorer cognitive and academic performance [59,60,61] and poorer neuropsychological performance during adulthood [62]; and health education policies for nutrition must promote seafood intake at these ages [28,49]. Various factors may contribute to the low frequencies of fish consumption in children and adolescents, including habit and unfamiliarity, and are mostly related to a low frequency of consumption in the home [11,63,64]. Negative attitudes to fish bones, the smell and appearance of fish and the perceived inconvenience and difficulty of preparing and cooking it also create barriers [25,40,64]. During childhood and adolescence in particular, the attitudes of friends [65] and parents [66] are influential when it comes to eating fish, since eating fish is rarely advertised in social networks and media, and it is considered an expensive option by some parents [67]. In our study we examined the influence of family and social aspects as determinants of seafood intake in children/adolescents. We found a significantly lower intake of some type of fish and the corresponding estimated DHA+EPA intake in children/adolescents whose mothers worked outside the home, and no such effect for fathers. A possible explanation is that mothers working outside the home have less time to prepare food, since, unfortunately, gendered division of labor persists within households as regards meal planning/preparation and food shopping [68,69]. A study in Taiwan related non-standard work schedules of the parents to the consumption of unhealthy food by the children, with more opportunities to eat snack foods or fast foods and for skipping breakfast [70].

In the patriarchal model of many families, the preparation of foods (cooking) by all the family’s members is more commonly undertaken by mothers than fathers. However, a study conducted on daily food choices among families in the United Kingdom showed that, even when it is considered an essential part of the diet along with the consumption of vegetables, fish consumption is deemed a complicated and time-consuming preparation, and often tends to be replaced by prepared foods or foods with easier and faster preparation requirements [71]. A Brazilian study assessing the mother-father gender difference in imitation of healthy food behavior by children found that in low income households (but not in high income households) an increase in the mother’s consumption of healthy food had a greater influence than the father’s on the children’s diet [72]. In our study, only 6.1% of the fathers were unemployed/househusbands, whereas this rate in mothers rises to 29.2%. It could be speculated that increased food-related work for women in larger households may partially be explained by the fact that women with younger children in the household may not work outside the home, and also because unemployment rates are higher for women in several European countries, including Spain [73]. However, with women′s increasing participation in the workforce, men have increased their contribution to household work and the gendering of food work is fortunately changing [68,69]. A recent prospective population-based nutritional study conducted on thousands of families in Scandinavia showed that the number of husbands cooking at home increased between 1997 and 2012, while the number of women cooking declined [68]. When explaining the socioeconomic disparities in children′s diets, several studies have showed that the home feeding environment for children of mothers with a low level of education was more limited than that of children of highly educated mothers [74,75,76]. In our study we found that children/adolescents whose mothers have a middle level of education (secondary school) have a lower estimated intake of DHA+EPA than children and adolescents whose mothers have primary school and university education. This apparent discrepancy regarding the protective role of parents’ high level of education on the healthy diet of children [77] could be explained by the higher rate of employment outside the home in women with low educational levels (primary school), rather than being at home and spending more time on cooking and preparing healthy food such as seafood. The differences between mothers with secondary school and university education can be explained by the fact that higher educational levels provide more knowledge of healthy food and nutritional aspects, because the percentages for unemployment and household duties in our study did not differ between mothers with middle levels of education (secondary school) and those with higher levels (university studies). Confirming the role of the mother’s high level of education in children’s nutrition, a recent study demonstrated that the children of mothers with a high educational level consumed more pieces of fruit and vegetables per day, and were more likely to have breakfast on a daily basis [38,78,79,80]. Likewise, a study of the influence of the family on children’s diet in Indonesia showed that 94% of mothers interviewed consumed fish during the dry season, and 79.7% of the children consumed a similar amount during the same period. In addition, children who consumed fish were five times as likely to have adequate dietary diversity. The strongest predictive factor for children’s adequate dietary diversity was mother’s adequate dietary diversity [81]. Another study showed that the variables that most increased fish intake in rural families were the size of the family and income, and in urban families variables were the size of the family, income and food preferences, with no major differences in behavior between the two environments. Mothers motivate families to consume fish because of its high nutrient content, and because it improves their offspring’s intelligence and reduces the risk of chronic diseases [82].

Future research could determine the influence of parents’ social aspects and gender differences on the types of foods consumed outside the home, ready-to-eat foods and frozen foods, in order to promote further exploration of these phenomena and the design of health education programs involving the whole family, in order to provide better nutrition for all family members. An American pilot study is assessing a dietary intervention in 60 low-income families aimed at reducing inequalities in health behaviors, based on mother-child dyads and training in healthy habits through videos, phone calls, printed materials, etc. This study could expand if successful and target preschool children, parents and teachers [26]. Our study has several limitations.

As regards limitations to this study, first data collection using questionnaires for food frequency intakes may contain errors due to inaccuracies in the amounts recorded and to their basis in reports from parents rather than children. However, we are confident that the information in the nutritional assessment was reliable, because the parents made a great deal of effort, showed considerable interest in the study from the beginning, and received training and support when responding to the food records. In addition, the rate of refusal to participate in the study was about 1%, and as such we consider our study has strong internal validity. A second limitation is represented by the fact that we did not estimate the intake of other omega-3 acids from other foods, but we trust in the major contribution of fish and seafood as a main source of EPA+DHA in the Spanish diet as well as in other countries [13,33,50]. Our study encourages health and education agencies to implement programs for parents and children regarding healthy dietary habits in Spain, including fish and seafood consumption. Given that fish consumption is the main source of dietary long chain omega-3 fatty acids in many countries, promoting fish consumption in a balanced diet and other positive eating behaviors is strongly desirable in our context in the future.

## Figures and Tables

**Table 1:** Characteristics of the study sample.

	Control (*n* = 87)
Age	9.9 ± 2.0 (range 4–13)
Gender	Female *n* = 52 (39.7%) Male *n* = 79 (60.3%)
Underweight	*n* = 31 (23.7%)
Normal weight	*n* = 45 (34.4%)
Overweight	*n* = 32 (24.4%)
Obese	*n* = 23 (17.6%)
Mother’s Educational level	Primary 25.2% Secondary 42.7% University 35.9%
Father’s Educational level	Primary 21.4% Secondary 37.4% University 37.4%
Mother’s work	Unemployed/housewife 29.2% Primary 0% Secondary 16.2% Tertiary 54.6%
Father’s work	Unemployed 6.1% Primary 4.6% Secondary 35.1% Tertiary 54.2%
Living with two parents	82.4%
Living with one parent	17.6%
